# Small Intestine-Targeted Long-Acting Oral Insulin Formulation Based on Engineered Milk Protein Nanoparticles

**DOI:** 10.1021/acsabm.5c02114

**Published:** 2025-12-24

**Authors:** Anbu Mozhi Thamizhchelvan, Yuancheng Li, Jonathan Padelford, Ce Yang, Chunhua Yang, Peijian He, Ashan Galhena, Tianhe Wu, Malgorzata Lipowska, Hui Mao

**Affiliations:** † Department of Radiology and Imaging Sciences, 12239 Emory University School of Medicine, Atlanta, Georgia 30322, United States; ‡ 1373 5M Biomed, LLC, Atlanta, Georgia 30303, United States; § Institute for Biomedical Sciences, Georgia State University, Atlanta, Georgia 30303, United States; ∥ Department of Medicine, 12239 Emory University School of Medicine, Atlanta, Georgia 30322, United States

**Keywords:** insulin, oral delivery, milk protein, nanoparticle, diabetes, long acting

## Abstract

Insulin therapies remain essential for glycemic control in diabetes mellitus, yet conventional subcutaneous injection is associated with poor patient compliance, risk of hypoglycemia, and other adverse effects. Oral insulin formulations offer a promising alternative by improving patient adherence and mimicking the endogenous insulin pathway. However, their clinical trials, mostly in enteric capsules and tablets, have yielded limited efficacy due to low insulin bioavailability. In this study, we prepared an oral insulin formulation by coencapsulating insulin and the permeation enhancer sodium caprate (C_10_) into milk protein casein-based nanocarriers (casNP). The casein shell was optimized for stable loading of insulin/C_10_, guided by *ex vivo* gut sac studies with different C_10_ concentrations. Reported casNP/insulin/C_10_ exhibited excellent stability in simulated gastric fluid and enabled insulin release in simulated jejunal fluid via trypsin-mediated casein degradation. Oral administration in mice resulted in a stomach half-emptying time of <15 min, small intestine delivery efficiency of 10.8 ± 1.7%, and insulin bioavailability of 18.1%, as measured in liver and plasma. Oral casNP/insulin/C_10_ (50 IU/kg) exhibited a blood sugar-lowering efficacy comparable to subcutaneously injected Insulin Aspart (5 IU/kg), while extending the duration of action by approximately 6 h and preventing hypoglycemia in fasted mice. These findings demonstrate that casNP/insulin/C_10_ is a promising oral insulin formulation for managing diabetes.

## Graphical abstract

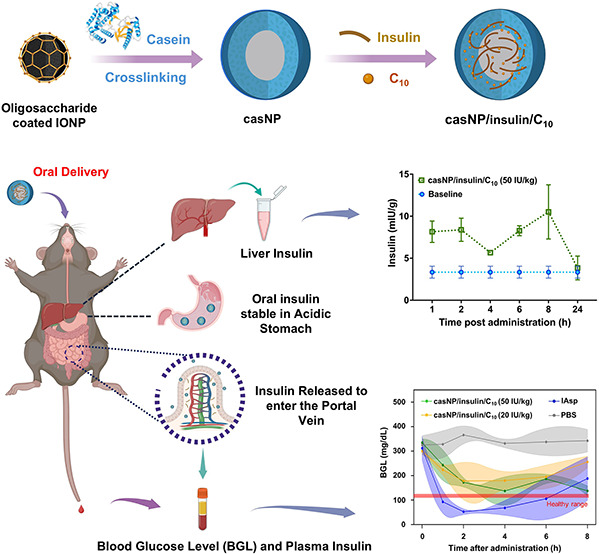

## 1 Introduction

Diabetes mellitus has become a global health challenge, affecting a large population of people worldwide. Currently, for patients suffering from type 1 diabetes (T1DM) and significant portions of patients with type 2 diabetes (T2DM), insulin therapy via subcutaneous (s.c.) administration through insulin pens or pumps remains a major pharmacological intervention for managing diabetic conditions and maintaining glycemic control. However, s.c. injection is not only inconvenient and causes discomfort but can also cause side effects, such as local infections. Thus, it is particularly difficult to comply with certain patient populations,^1,2^ such as pediatric patients. Furthermore, s.c. administration delivers insulin with suboptimal pharmacokinetics (PK) and pharmacodynamics (PD), which contribute to risks of side effects from long-term use of insulin injections, including hypoglycemia, peripheral hyperinsulinemia,^3^ lipodystrophy,^4^ weight gain,^5^ and hypoglycemia-associated autonomic failure (HAAF).^6^ Therefore, oral insulin has been considered as a paradigm-shifting solution in diabetes management for decades. The convenience of an oral formulation can substantially improve patient adherence and care delivery to a wide range of patient populations. More importantly, once orally delivered insulin reaches and is absorbed by the targeted section of the gastrointestinal (GI) tract, it mimics the physiological route of endogenous insulin secretion through the hepatic portal circulation.^7,8^ Absorption of insulin by the GI tract promises enhanced safety and improved PK/PD profiles that can minimize peripheral hyperinsulinemia and reduce adverse effects such as weight gain and hypoglycemia, as demonstrated in recent studies.^9–11^

Despite these compelling benefits and clinical needs, the development of oral insulin has been hindered by formidable biological and technological barriers, including rapid enzymatic degradation in the acidic stomach and limited intestinal permeability, leading to extremely low bioavailability (<1%).^11,12^ While several clinical trials are underway for new oral insulin formulations, none have yet become commercially available, as highlighted by the recent discontinuation of the Phase III trial of ORMD-0801 (NCT04606576 and NCT04754334) and the unsatisfactory outcomes of Tregopil (NCT03430856).^13,14^ Nevertheless, there is continuous effort to pursue and apply novel approaches and new materials for oral insulin formulations. For instance, zwitterionic micelles,^15–17^ quantum dots with a chitosan/glucose polymer coating,^18^ polyester nanoparticles,^19^ metal–organic framework (MOF) nanoparticles,^20,21^ dendritic mesoporous silica nanoparticles,^22^ and dendrimer nanocomplexes^23^ have been studied. These formulations demonstrated promising potential as carriers for oral insulin delivery with featured properties and functionalities, such as protecting insulin from gastric degradation, enabling triggered release under specific physiological conditions, and enhancing intestinal uptake via transcellular or paracellular pathways.^24^

Iron oxide nanoparticles (IONPs) are widely used in biomedical applications due to their magnetic properties, biocompatibility, and potential to provide a multifunctional platform for imaging and drug delivery.^25^ Coating IONPs with oligosaccharides enhances their stability and provides functional sites for further surface modification.^26^ Milk protein casein, representing approximately 80% of bovine milk, is a natural product with excellent biocompatibility, pH-responsive behavior, and a natural function as a nutrient carrier.^27,28^ Casein consists of four phosphoprotein components (αs1-, αs2-, β-, and κ-casein) that spontaneously self-assemble into micellar nanostructures with hydrophobic cores and hydrophilic shells. The formed casein micelles can encapsulate therapeutic molecules via hydrophobic, electrostatic, or covalent interactions,^27,29^ while protecting payloads from gastric acidity and enzymatically triggered release in the gastrointestinal environment, which are properties sought for oral delivery systems.^28,30^

Here, we introduce a new fast- and long-acting liquid-form oral insulin formulation utilizing the natural milk protein casein to form a core–shell nanocomposite (casNP) to codeliver insulin and the permeation enhancer sodium caprate (C_10_) to the preferred small intestine for optimal absorption and glycemic control. Milk protein casein forms a micelle-like outer layer on the core of the oligosaccharide-coated iron oxide nanoparticle (IONP) via cross-linking, as we demonstrated previously.^26,31^ The casein outer layer of casNP maintains the properties of the milk protein as a natural carrier for nutrients due to its porous structure.^32^ In addition, it is stable at low pH and resistant to proteases in the acidic stomach, allowing for targeted delivery of insulin and C_10_ to the small intestine for rapid enzymatic release of payloads to achieve fast insulin action. We report the preparation of insulin and C_10_-loaded casNP (casNP/insulin/C_10_) as well as characterizations of its properties. The small intestine-targeted delivery and triggered release of casNP/insulin/C_10_ were investigated *in vitro* and *in vivo*, followed by an evaluation of insulin bioavailability and glycemic control efficacy of orally administered casNP/insulin/C_10_ in a diabetic mouse model.

## 2 Materials and Methods

### 2.1 Materials

Casein from bovine milk (C3400–500G), C_10_ (C4151–5G), and fluorescein isothiocyanate (FITC)-labeled insulin (I3661–5MG) were purchased from Sigma-Aldrich (St. Louis, MO, USA). Human recombinant insulin (0219390080) was obtained from MP Biomedicals (Irvine, CA, USA). Insulin aspart (IAsp, Y0000349) was purchased from EDQM (Strasbourg, France). Trypsin (0.25%, SH30042.01) was ordered from Hyclone (South Logan, Utah, USA). Pepsin (10108057001) was purchased from Roche Diagnostics (Germany). Streptozotocin (STZ, AG-CN2–0046-G001) was obtained from AdipoGen Life Sciences (San Diego, CA, USA). Insulin ELISA kit (ab100578) and anti-E-cadherin antibody (ab231303) were purchased from Abcam (Waltham, MA, USA). Iron nitrate nonahydrate (216828–100G), sodium oleate (O7501–1G), d-(+)-glucose (G8270–100G), IR-783 (543292–250MG), 4-mercaptobenzoic acid (706329–1G), *N,N’*-disuccinimidyl carbonate (8149710005), nickel perchlorate hexahydrate (309338–5G), glutaradehyde (G6257–100 ML), EDTA-free protease inhibitor (4693159001), PHOSSTOP (4906837001), citric acid (251275–100G), trifluoroacetic acid (TFA, 302031–100 ML), *o*-nitrophenol (241326–50G), 1,10-phenanthroline monohydrate (320056–5G), sodium hydroxide (221465–500G), nitric acid 67–70% (NX0407), 2mMercaptoethanol (M3148), deuterium oxide containing 0.75 wt % 3-(trimethylsilyl)­propionic-2,2,3,3-d4 acid (293040–25G), hexane (293252–4L), chloroform (319988–4 × 4L), dimethylformamide (DMF, 227056–1L), methanol (MX0487–5), Amicon ultra centrifugal filter with 30 kDa MWCO (UFC8030), Immobilon Western chemiluminescent HRP substrate (WBKLS0500), and bovine serum albumin (BSA, A7906–50G) were purchased from Sigma-Aldrich (St. Louis, MO, USA). Bicinchoninic acid (BCA) protein assay kit (PI23227), goat antimouse IgG (H + L) secondary antibody (A-21422), RIPA buffer (PI89901), 1.3 mL EDTA K3 microtube (NC9414041), and 1-octadecene (129310010) were purchased from Thermo Fisher Scientific (Waltham, MA, USA). Phospho-Akt (Ser473) antibody (9271), glyceraldehyde 3-phosphate dehydrogenase (GAPDH-14C10) rabbit monoclonal antibody (2118), and goat antirabbit IgG H&L (HRP) antibody (7074) were obtained from Cell Signaling Technology (Danvers, MA, USA). Precast polyacrylamide gel (10%, 4568034), 2 × Laemmli buffer (1610737), Precision Plus Protein Kaleidoscope standards (1610375), poly­(vinylidene fluoride) (PVDF) membranes (1620177), and 10 × Tris/glycine/SDS (1610732) were obtained from Bio-Rad (Hercules, CA, USA). Ethanol (200 proof, V1001) was purchased from Decon Laboratories Inc. (King of Prussia, PA, USA). PES syringe filters with 0.2 μm pore membrane (431222) were purchased from Corning Inc. (Corning, NY, USA). Phosphate-buffered saline (MRGF-6235) was purchased from Growcells (Irvine, CA, USA). Tris-buffered saline (TBS, pH 7.4, 351–086–101) was purchased from Quality Biological (MD, USA). Mayer’s hematoxylin solution modified (786–1263) was ordered from G Biosciences (St. Louis, MO, USA). Eosin Y stain 1% alcoholic (7111) was obtained from Richard-Allan Scientific (Kalamazoo, MI, USA). Permount mounting medium (17986–01), fluoro- gel with Tris Buffer (17985–10), and carbon-coated copper grids (CF300-Cu-50) were purchased from Electron Microscopy Sciences (Hatfield, PA, USA). All materials were used as received.

### 2.2 Synthesis of CasNP

CasNP was prepared following our method published earlier.^26,31^ First, iron nitrate nonahydrate and sodium oleate were reacted in a mixed solvent consisting of deionized (DI) water, hexane, and ethanol to yield iron-oleate precursors. These precursors were then heated to 325 °C for 10 and 30 min to obtain oleic acid-capped IONPs with core diameters of 5 and 20 nm, respectively. Oligosaccharide-coated IONPs were then synthesized by adding oleic acid-capped IONPs dropwise into a glucose solution in DMF, preheated to 90 °C. The reaction solution was then heated to 120 °C for 3 h and cooled to room temperature before the oligosaccharide-coated IONPs were precipitated by adding an excess amount of ethanol. The coated IONPs were then collected by centrifugation and redispersed in DI water for further coating with caseins.

To make the casein outer layer on IONP, the saturated casein solution in 0.2 M NaOH was prepared before mixing with oligosaccharide-coated IONPs at a mass ratio of 1:5. The mixture was kept on a shaker at room temperature for 18 h to allow the nonspecific adsorption of casein on the surface of IONP. Afterward, 0.4% glutaraldehyde was added to the solution in a glutaraldehyde/casein weight ratio of 1:500 to cross-link casein molecules. After 2 h, the reaction solution was filtered using an Amicon centrifuge filter with 30 kDa MWCO to remove free caseins and unreacted glutaraldehyde. CasNP was collected and washed three times with DI water for further use.

### 2.3 Preparation of CasNP/Insulin/C_10_

Loading insulin and C_10_ on casNP to make casNP/insulin/C_10_ was accomplished via encapsulation, since the cross-linked casein outer layer remains porous and has a high affinity for both the insulin peptide and the small molecule C_10_. Briefly, casNP (1 mg/mL in water) was mixed with insulin and C_10_ in a mass ratio of 1:1:5 (casNP:insulin:C_10_). The mixture was kept inverted on a shaker at room temperature overnight. Afterward, unloaded insulin and C_10_ were removed by filtering the mixture using an Amicon centrifuge filter with a 30 kDa MWCO. Collected casNP/insulin/C_10_ was washed with DI water three times and reconstituted in DI water with a concentration of 1000 IU/mL. The concentrations of remaining insulin and C_10_ in the filtrates were measured using an Ultimate 3000 HPLC system (Thermo Fisher Scientific, Waltham, MA, USA) with *o*-nitrophenol added as the internal standard. Acetonitrile/water (30:70, v/v) at pH 2.4 (adjusted using TFA) was used as the mobile phase, with a flow rate of 1 mL/min. The detection wavelength was set at 225 nm. The column was equilibrated for at least 15 min using the mobile phase before measurement. The amount of unloaded C_10_ was cross-validated using nuclear magnetic resonance (NMR) spectroscopy. Briefly, the filtrate was concentrated using a rotary evaporator (Buchi Corporation, New Castle, DE, USA). The residue was dissolved in deuterium oxide with 0.75 wt % 3-(trimethylsilyl) propionic-2,2,3,3-d4 acid as the internal reference. Quantification of C_10_ was based on the alkyl peaks between 1.0 and 1.8 ppm in the NMR spectra. The amounts of loaded insulin and C_10_ were calculated by subtracting the amounts of unloaded insulin and C_10_ from the amounts added. The loading efficiencies and encapsulation efficiencies of insulin and C_10_ were calculated based on eqs 1 and 2:

1 $${\mathrm{Loading}\;\mathrm{efficiency}=M}_{\mathrm{payload}}{/M}_{\mathrm{casNP}}\times 100\%$$

2 $${\mathrm{Encapsulation}\;\mathrm{efficiency}=M}_{\mathrm{encap}}{/M}_{\mathrm{added}}\times 100\%$$

where *M*_payload_ is the mass of encapsulated insulin or C_10_, *M*_casNP_ is the mass of casNP, and *M*_encap_ and *M*_added_ are the masses of encapsulated and added insulin or C_10_, respectively.

The loading ratio of insulin/C_10_ for effective insulin penetration through the small intestine was determined using everted gut sacs prepared following published methods.^33,34^ Briefly, freshly collected mouse jejuna were placed in an ice-cold NaCl Ringer solution (50 mL) and cut into parts approximately 4 cm in length. The jejunum parts were tied at one end with a suture and gently everted over a capillary tube. The everted jejuna were then filled with 200 μL of Ringer solution and sealed by tying the other end with a suture. In 2 mL centrifuge tubes, the prepared gut sacs were submerged in 1 mL PBS containing 0.5 mM insulin (83.5 IU/mL) with C_10_ concentrations ranging from 0 to 0.1 mM. The tubes were placed in an H5000-HC MultiTherm heating shaker (Benchmark Scientific, Sayreville, NJ, USA) at a speed of 600 rpm at 37 °C for 15 to 45 min. Afterward, 500 μL of the PBS solution was taken from each tube and mixed with *o*-nitrophenol (2 mg/mL in DI water, 500 μL) to quantify the residual insulin in the solution using a Thermo Scientific Ultimate 3000 HPLC system with *o*-nitrophenol as the internal standard.^35,36^ In addition, the gut sacs were embedded in the O.C.T. compound and stored at −80 °C until further immunofluorescence staining (Section 2.9).

For imaging-based biodistribution and histological validation experiments, we also labeled casNP/insulin/C_10_ with the near-infrared (NIR) dye for fluorescence imaging. In this case, NHS-NIR830 dye molecules (excitation 791 nm, emission 810 nm) were synthesized from IR-783 following our published method^37^ and then conjugated to the NH_2_ groups of caseins by reacting with casNP/insulin/C_10_ in PBS for 2 h at room temperature. The conjugation was validated by the emerging absorption at 791 nm in the UV–vis spectrum by using a Thermo Scientific GENESYS 150 spectrometer (Waltham, MA, USA).

### 2.4 Characterization of Physical Properties for CasNP/Insulin/C_10_

Transmission electron microscopy (TEM) was used to determine the core size and visualize the morphology of the casNP and casNP/insulin/C_10_. Samples were prepared by dropping diluted solutions on the carbon-coated copper grid for air-drying overnight and then examined on a Hitachi H-7700 microscope (Santa Clara, CA, USA, accelerating voltage: 80 kV). For negatively stained electron microscopy, casNP/insulin/C_10_ were adsorbed onto freshly glow-discharged, carbon-coated copper grids for 5 min. Excess fluid was carefully removed by blotting the grid surface with filter paper. The grids were then incubated, sample side down, on a drop of 2% uranyl acetate for 1 min, followed by blotting to remove excess stain. Imaging was performed using a JEOL JEM-1400 microscope (Peabody, MA, USA) operated at 80 kV. Electron micrographs were acquired by using a 2048 × 2048 charge-coupled device (CCD) camera (UltraScan 1000, Gatan Inc., Pleasanton, CA, USA).

Averaged hydrodynamic diameters and zeta potentials of casNP and casNP/insulin/C_10_ were measured on a Zetasizer Nano S90 (Malvern, Westborough, MA, USA) and averaged based on three independent measurements. The ultraviolet–visible (UV–vis) spectra of oligosaccharide-coated IONP, casNP, and casNP/insulin/C_10_ were recorded using a Thermo Scientific GENESYS 150 spectrometer (Waltham, MA, USA). Fe contents of casNP and casNP/insulin/C_10_ were quantified using a 1,10-phenanthroline colorimetric assay following the established protocol.^38–40^ CasNP and casNP/insulin/C_10_ were briefly digested in concentrated nitric acid to break down IONPs into Fe^3+^, which was then reduced to Fe^2+^ using hydroquinone to form the Fe^2+^ complex with 1,10-phenanthroline. The characteristic absorbance of the complex at 508 nm was measured to quantify Fe concentrations. The casein content of casNP was measured by a BCA protein assay kit following the manufacturer’s instructions, with oligosaccharide-coated IONPs at the same Fe concentration used as the baseline control.

### 2.5 Stability and Release Profile of CasNP/insulin/C_10_ in Simulated Gastric and Intestinal Fluids

Sodium dodecyl sulfate-polyacrylamide gel electrophoresis (SDS-PAGE) was used to analyze the stability and breakdown of the casein coating on casNP/insulin/C_10_ in simulated gastric and intestinal fluids. CasNP/insulinC_10_ was allowed to briefly digest in simulated gastric fluid (1.0 mg/mL pepsin^41,42^ in McIlvaine buffer at pH 2.2),^43^ duodenal fluid (0.02 mg/mL trypsin^44,45^ in McIlvaine buffer at pH 6.0),^43^ and jejunal fluid (0.7 mg/mL trypsin^46,47^ in McIlvaine buffer at pH 7.0) for 15, 30, and 60 min, respectively. At each time interval, the reaction was stopped by the addition of 1 M sodium bicarbonate. After electrophoresis, the gel was washed with DI water three times, for 5 min each time. The gel was then fixed with 20% methanol for 30 min, shaken, and washed with plenty of DI water, followed by staining with Coomassie Brilliant Blue solution overnight. After washing three times (5 min each time) with DI water, the gel was imaged using a ChemiDoc MP Imaging System (Hercules, CA, USA). The protein bands were analyzed and quantified using ImageJ software (NIH, Bethesda, MD, USA).

For studying insulin release, we prepared casNP/insulin/C_10_ with FITC-labeled insulin. The casNP/FITC-insulin/C_10_ (1000 IU/mL) was incubated in simulated gastric fluid for 15, 30, and 60 min. Filtration using an Amicon Ultra centrifugal filter with 100 kDa MWCO was then performed to collect the casNP/insulin/C_10_. We measured changes in the hydrodynamic diameters of treated casNP/insulin/C_10_ formulations compared with their untreated counterparts to assess product stability. The casNP/FITC-insulin/C_10_ collected after the 60 min treatment in gastric-mimicking conditions was further treated with simulated jejunal fluid containing 0.7 mg/mL trypsin for 15, 30, 60, and 120 min. The digested casNP/insulin/C_10_ were then collected for measuring hydrodynamic sizes after centrifugal filtration using an Amicon filter with 100 kDa MWCO. The filtrates were collected for measuring the release of FITC-insulin on a PerkinElmer LS 55 fluorescence spectrometer (Waltham, MA, USA) with excitation/emission set at 488/525 nm. Contents of released insulin were normalized to the total loaded FITC-insulin as 100%. All experiments were performed three times.

### 2.6 Diabetic Animal Model

The diabetic mouse model was prepared based on literature-reported procedures.^48^ All animal experiments were approved by the Institutional Animal Care and Use Committee (IACUC) of Emory University under protocol PROTO202100141. The animals were housed with a 12 h light/12 h dark cycle and had *ad libitum* access to food and water. Briefly, male C57BL/6 mice (6–8 weeks old) were fasted overnight with free access to water before receiving intraperitoneal injections of streptozotocin (STZ) at a dose of 100 mg/kg body weight for 3 consecutive days. STZ was freshly prepared in a 10 mM citrate buffer (pH 4.5) before each injection to maintain stability. The supply of food and water was resumed for the mice after the STZ injection. Blood glucose levels (BGL) were monitored daily using a glucometer (True Metrix Air, Trividia Health, Inc.) by blood sampling from a tail snip. The diabetic condition was set and confirmed when measured BGL exceeded 300 mg/dL for 3 consecutive days.

### 2.7 Biodistribution of CasNP/Insulin/C_10_ in Mice

Diabetic mice were given an oral gavage of NIR830-labeled casNP/insulin/C_10_ at a dosage of 50 IU/kg body weight and euthanized at 0.25, 1, 2, 4, 6, 8, and 24 h postadministration (*n* = 3 per time point) to collect major organs, including the liver, spleen, kidneys, stomach, and intestine. *Ex vivo* fluorescence imaging of the collected organs was performed to investigate the distribution of NIR830 signals using an *In Vivo* Imaging Spectrum (IVIS) system (PerkinElmer, Waltham, USA) with a field of view (FOV) of B level and an exposure time of 1 ms. The organs were arranged on a black plastic board to minimize background interference. Radiance efficiencies of different organs were analyzed using Living Image Software (PerkinElmer, Waltham, USA) based on region-of-interest (ROI) measurements. A small piece (∼1 cm in length) of NIR-positive small intestine tissue was collected from each mouse for pathological and immunofluorescence staining. Afterward, the organs were weighed and lyophilized before being digested in concentrated nitric acid at 70 °C overnight. Fe concentrations of the digested organs were measured using the 1,10-phenanthroline colorimetric assay as described above. Each sample was measured in triplicate. The Fe contents of the organs were presented as mg Fe/g organ weight.

For quantifying insulin in the plasma and liver, diabetic mice were randomly divided into three groups (*n* = 5 per data point) to receive casNP/insulin/C_10_ at 20 and 50 IU/kg body weight via oral gavage and IAsp at 5 IU/kg body weight via s.c. injection. Mouse blood was collected via cardiac puncture at 1, 2, 4, 6, 8, and 24 h postadministration, and transferred to an EDTA K3 microtube. The microtubes were centrifuged at 600 × *g* for 10 min at 4 °C to harvest the plasma. Mice receiving no treatment were used as the baseline control. Mouse liver tissues were dissected on ice as quickly as possible upon collection to prevent protease degradation. A small piece of liver tissue from each mouse was stored separately for Western blots (2.8). The remaining tissue was weighed, placed in 5 mL Eppendorf microfuge tubes, and stored at −80 °C for later use or kept on ice for immediate homogenization. PBS (2 mL) was added to the tube prior to the homogenization of liver tissues using an OMNI International Tissue Master 125 homogenizer (Kennesaw, GA, USA). The tissue extracts were then centrifuged at 15000 × *g* for 10 min at 4 °C to collect the supernatant. The insulin concentrations in the supernatant and plasma were measured using an enzyme-linked immunosorbent assay (ELISA) kit following the manufacturer’s instructions.

For measuring the plasma insulin levels in fasted diabetic mice, we randomly divided mice fasted overnight into two groups, with one receiving casNP/insulin/C_10_ orally at 50 IU/kg body weight and the other receiving s.c. injection of IAsp at 5 IU/kg body weight. All mice were kept fasted until blood collection via cardiac puncture at 1, 2, 4, 6, and 8 h after administration (*n* = 5 per group per time point). Measurements of insulin levels in the plasma were carried out using an ELISA kit as described above.

### 2.8 Examination of Akt Phosphorylation Using Western Blot

Collected liver tissues (Section 2.7) were immediately frozen in dry ice, transferred to a RIPA buffer cocktail containing protease and phosphatase inhibitors, and homogenized using an OMNI International Tissue Master 125 homogenizer (Kennesaw, GA, USA). The homogenized samples were then centrifuged at 14,000 × *g* for 20 min at 4 °C to collect the supernatant. The total protein concentration in each sample was quantified using a BCA protein assay kit following the manufacturer’s instructions, before each sample was diluted appropriately with RIPA buffer cocktail to ensure equal protein concentrations across all groups. The samples were then mixed with 2 × Laemmli buffer and β-mercaptoethanol before loading onto 10% polyacrylamide gels. The SDS-PAGE-separated proteins were subsequently transferred to PVDF membranes, which were washed with 1× Tris-glycine buffer containing 20% (v/v) methanol before being blocked with 3% BSA in 1× TBS containing 0.1% (v/v) Tween 20 (TBST) for 1 h at room temperature on an orbital shaker. The membranes were then incubated with primary antibodies against rabbit anti-pAkt (Ser473) at a 1:1000 dilution in TBST containing 3% BSA overnight at 4 °C. GAPDH was used as a loading control, with its primary antibody also diluted to 1:1000 in the blocking buffer. Afterward, the membranes were washed with TBST and incubated with horseradish peroxidase (HRP)-conjugated goat antirabbit secondary antibody (1:5000 dilution in TBST) at 37 °C for 1 h. Following another wash with TBST, the membranes were incubated in Immobilon Western chemiluminescent HRP substrate for 1 min. Protein bands were visualized using a ChemiDoc MP Imaging System (Hercules, CA, USA) and analyzed using ImageJ software (NIH, Bethesda, MD, USA).

### 2.9 Histology and Immunofluorescence Staining of Small Intestine Tissues

To examine the morphology of the small intestine tissues and the distribution of FITC-insulin and NIR830-labeled casNP/insulin/C_10_, hematoxylin–eosin (H&E) and immunofluorescence staining were performed on the collected gut sacs and small intestines. The frozen O.C.T.-embedded intestine tissue samples were sectioned into 7 μm thick slices using a CM1900 cryostat microtome (Leica, Wetzlar, Germany). The sections were fixed in 100% acetone for 10 min, then air-dried before being stored at −20 °C.

Hematoxylin–eosin (H&E) staining was carried out following our published protocol.^40^ Immunofluorescence staining was carried out by washing tissue sections with 1 × PBS for 20 min, and blocking the sections with 10% goat serum (dissolved in PBS + 0.1% Tween) for 1 h at room temperature to minimize nonspecific binding. The samples were then incubated with the anti-E-cadherin antibody (1:200 dilution in PBS with 1% BSA) at 4 °C overnight. Tissue sections were then washed three times with PBS and incubated with a secondary antibody (goat antimouse IgG 555) for 1 h at room temperature. Afterward, the sections were washed with PBS and stained for nuclei with Hoechst. Slides were further washed three times with PBS, mounted with fluoro-gel, and sealed with coverslips. Histology and fluorescence imaging of the stained tissue sections were performed by using a Revolve fluorescence microscope (Discover Echo Inc., San Diego, CA, USA).

### 2.10 Pharmacokinetic and Efficacy Evaluation of Oral CasNP/Insulin/C_10_ versus Subcutaneous Insulin in Nondiabetic Mice

For the pharmacokinetic assessment, four male and four female mice were used. Two male and two female mice received oral casNP/insulin/C_10_ at a dose of 50 IU/kg, while two male and two female mice received subcutaneous (s.c.) insulin at 5 IU/kg. Whole-blood samples (∼20 μL) were collected from the tail snip at baseline (0 min) and at 15, 30, and 60 min, as well as 2, 4, and 8 h postadministration. Insulin concentrations in whole-blood samples were measured using a human insulin ELISA kit according to the manufacturer’s instructions. The relative insulin bioavailability of casNP/insulin/C_10_ was calculated based on the formula below:

3 $$\mathrm{Bioavailability}=\left({\mathrm{AUC}}_{\mathrm{casNP}/\mathrm{insulin}/C10}\times {\mathrm{dose}}_{\mathrm{insulin}}\right)/\left({\mathrm{AUC}}_{\mathrm{insulin}}\times {\mathrm{dose}}_{\mathrm{casNP}/\mathrm{insulin}/C10}\right)$$

where AUC_casNP/insulin/C10_ and AUC_insulin_ represent the area under the curve for the casNP/insulin/C_10_ and s.c. insulin profiles, and dose_casNP/insulin/C10_ and dose_insulin_ are the injected dosages of casNP/insulin/C_10_ and s.c. insulin, respectively.

### 2.11 Efficacy of CasNP/Insulin/C_10_ in Glycemic Control in Diabetic Mice

Diabetic mice were randomly divided into four groups (*n* = 5 per group) to receive casNP/insulin/C_10_ at 20 and 50 IU/kg body weight through oral gavage, IAsp at 5 IU/kg body weight via s.c. injection, and PBS as a placebo once daily at 8:30 am for 5 consecutive days. CasNP/insulin/C_10_ and IAsp were prepared as solutions in sterile water with concentrations of 30 and 7.5 IU/mL, respectively, which gave rise to administration volumes of 20 μL for casNP/insulin/C_10_ at 20 IU/kg body weight and IAsp, and 50 μL for casNP/insulin/C_10_ at 50 IU/kg body weight. PBS (sterile) was given via oral gavage at a volume of 50 μL. On Days 1, 2, and 5 after treatment, BGLs were measured at baseline (0 h, immediately before treatment) and at 1, 2, 4, 6, and 8 h post-treatment using a glucometer via tail snip, while the mice were kept fasted. Normal feeding resumed afterward. The mice were monitored for activities for two more hours. To evaluate the risk of hypoglycemia for casNP/insulin/C_10_, diabetic mice were fasted overnight and randomly divided into four groups (*n* = 5 per group) to receive the treatments as described above.

### 2.12 Hematological and Biochemical Analysis

Mice receiving casNP/insulin/C_10_ or PBS (*n* = 3 per group) were anesthetized 2 h after the treatment for retro-orbital blood collection. Collected whole blood (100 μL) was analyzed using a Vetscan VS2 chemistry analyzer for the levels of albumin (ALB), alkaline phosphatase (ALP), alanine aminotransferase (ALT), amylase (AMY), total bilirubin (TBIL), blood urea nitrogen (BUN), calcium (Ca), phosphorus (PHOS), creatinine (CRE), sodium (Na^+^), potassium (K^+^), total protein (TP), and globulin (GLOB). The hematology was assessed on a Vetscan HM5 animal hematology analyzer (Zoetis, Parsippany-Troy Hills, NJ, USA) using 50 μL of whole blood. Examined hematological parameters included the counts of white blood cells (WBC), lymphocytes (LYM), monocytes (MON), neutrophils (NEU), red blood cells (RBCs), hemoglobin (HGB), hematocrit (HCT), mean corpuscular volume (MCV), mean corpuscular hemoglobin (MCH), mean corpuscular hemoglobin concentration (MCHC), and platelets (PLT). All measurements were performed following the manufacturer’s guidelines.

### 2.13 Statistical Analysis

All data are presented as mean ± standard deviation. A two-way analysis of variance (ANOVA), followed by Tukey’s HSD posthoc test, was performed for multiple group comparisons. All statistical analyses were conducted using GraphPad Prism 9. Statistical significance was indicated as follows: *p < 0.05, **p < 0.01, ***p < 0.001, and ****p < 0.0001, while “ns” denotes no significant difference.

## 3 Results

### 3.1 Determination of the CasNP/Insulin/C_10_ Formulation

As illustrated in Figure 1A, casNP carriers are constructed by cross-linking the casein molecules that are nonspecifically adsorbed on the surface of oligosaccharide-coated IONPs. Insulin and C_10_ were then encapsulated in the porous casein layer of casNP.^32^ We prepared different casNPs by changing the IONP core diameters from 5 to 20 nm and the thickness of the casein layers (Table S1). After evaluating the monodispersity and colloidal stability of casNP using hydrodynamic sizes and the payload capacity controlled by casein contents and layer thickness, the casNP with the IONP core of 5 nm, hydrodynamic diameter of 23.3 ± 1.9 nm, and casein content of 81 ± 14% (weight%) was selected for making insulin- and C_10_-loaded casNP/insulin/C_10_ (Figure S1). TEM imaging further confirmed that the selected casNP was uniformly dispersed (Figure S1A).

**Figure 1:**
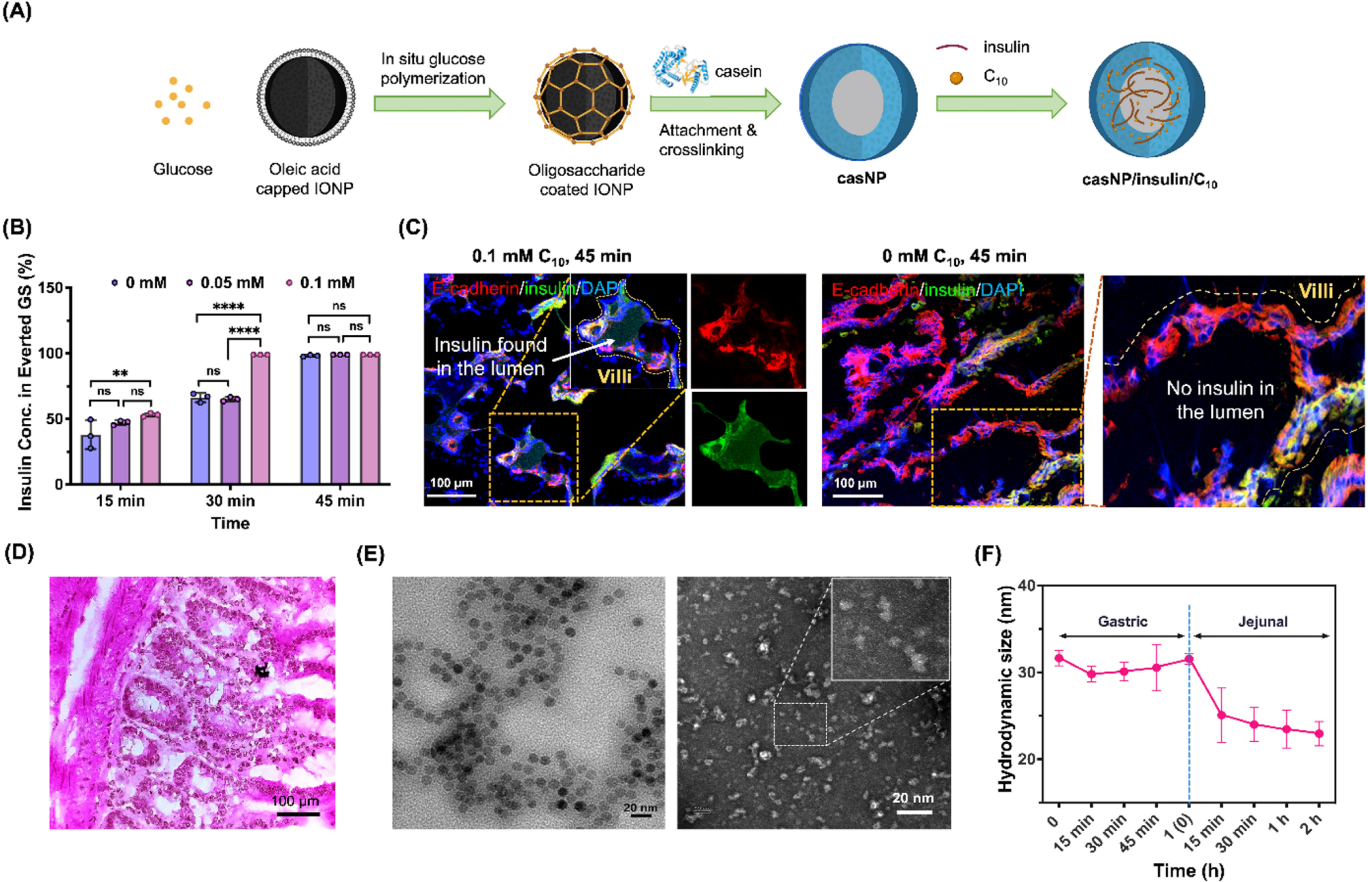
Preparation and characterization of casNP/insulin/C_10_ formulation with high insulin absorption by everted gut sacs. (**A**) Schematic illustration of the preparation of casNP and casNP/insulin/C_10_ with a core of oligosaccharide-coated IONP. (**B**) Efficiencies of insulin absorption by everted gut sacs with different incubation times and C_10_ concentrations. (**C**) Representative fluorescence images of the gut sacs after incubating with 0.5 mM insulin at 37 °C in the presence of 0 and 0.1 mM C_10_. E-cadherin was immunofluorescence stained with antibodies. (**D**) A representative image of H&E-stained gut sac after incubating with 0.5 mM insulin and 0.1 mM C_10_. (**E**) Representative TEM images of casNP/insulin/C_10_ without and with negative staining. Scale bar: 20 nm. (**F**) Hydrodynamic sizes of casNP/insulin/C_10_ in gastric and jejunal mimicking conditions for 1 and 2 h, respectively.

We then used everted gut sacs to investigate the optimal loading ratio of insulin and C_10_ for casNP/insulin/C_10_ based on the efficiency of C_10_-facilitated insulin absorption by the sacs. We first tested the insulin absorption of the sacs without the permeation enhancer C_10_. HPLC measurement of free insulin in the solution revealed that the insulin absorption by the sacs was time-dependent, with ∼100% absorbed after 45 min (Figure 1B). This baseline absorption likely results from passive diffusion and limited transcytosis of insulin across the intestinal epithelium, reflecting the intrinsic permeability of the gut tissue in the *ex vivo* sac model. In the presence of C_10_, the absorption was accelerated, reaching 100% absorption in 30 min, likely due to C_10_-facilited insulin permeation through the transient opening of tight junctions. To validate whether absorbed insulin simply attached to the surface of the villi or entered the villus lumen, we examined the distribution of FITC-insulin using fluorescence imaging. Figure 1C shows the presence of FITC signals, rising from FITC-insulin, inside the villi with a disrupted epithelium lining revealed by the immunofluorescence-stained E-cadherin after the sacs were treated with FITC-insulin and 0.1 mM C_10_. These results suggest the opening of tight junctions of the intestinal epithelium and the diffusion of FITC-insulin into the lumen of villi. In comparison, when C_10_ was not used, the FITC signals were mostly detected on the surface but not in the lumen of villi, with a continuous lining of E-cadherin. Taken together, the results indicated that C_10_ with the molar ratio of 1:5 to insulin, effectively promoted insulin permeation into the villi, which may enhance the efficiency of insulin absorption by the capillaries inside the villi to enter the portal vein and therefore improve the insulin bioavailability. H&E staining of representative gut sac sections revealed no observable structural damage to the small intestinal tissue after incubation with 0.5 mM insulin and 0.1 mM C_10_ (Figure 1D).

Based on the insulin-to-C_10_ weight ratio of ∼150:1 (equivalent molar ratio of 5:1), we prepared casNP/insulin/C_10_ with loading efficiencies of 23.8 ± 0.6% and 0.16 ± 0.02% for insulin and C_10_, respectively. The encapsulation efficiencies were measured to be 83.3 ± 2.2% and 69.1 ± 3.0% for insulin and C_10_, respectively. The encapsulation efficiency of insulin by casNP is higher than other nanoformulations, such as polymeric nanocapsules^49^ and lipid nanoparticles,^50^ suggesting that casNP with natural casein proteins is more efficient and therefore more cost-effective than other engineered nanocarriers in encapsulating insulin. After loading insulin and C_10_, casNP/insulin/C_10_ remained monodispersed with an average diameter of 14.2 ± 3.6 nm, as measured from TEM with and without negative staining (Figure 1E). The hydrodynamic size, zeta potential, and UV–visible spectrum of casNP/insulin/C_10_ did not exhibit significant differences compared to those of casNP (Figure S1B– D), indicating that the loading of insulin and C_10_ did not alter the physical properties of the casNP carriers.

The prepared casNP/insulin/C_10_ were then investigated for gastric stability *in vitro* by incubating casNP/insulin/C_10_ in simulated gastric fluid (1.0 mg/mL pepsin, pH 2.2) for 15, 30, 45, and 60 min. The hydrodynamic sizes of casNP/insulin/C_10_ measured by dynamic light scattering (DLS), did not show a significant change at any time interval (Figure 1F), indicating that casNP/insulin/C_10_ was resistant to aggregation in acidic conditions and degradation by protease in the stomach. It is also worth noting that natural caseins are prone to the hydrolysis of κ-casein subunits in the stomach, which exposes the internal hydrophobic subunits and forms insoluble curds with prolonged gastric retention (2–8 h).^51–53^ Our results indicated that the core–shell structure of casNP/insulin/C_10_ enabled the application of natural caseins in efficient small intestine-targeted drug delivery. Once exposed to simulated jejunal fluid (0.7 mg/mL trypsin, pH 7.0), the hydrodynamic size of casNP/insulin/C_10_ decreased from 35.1 ± 0.6 nm to 25.1 ± 3.1 nm within 15 min and further to 22.9 ± 1.4 nm after 2 h of incubation as a result of the casein layer being broken down by trypsin, a protease present in the pH-neutral condition of the intestine (Figure 1F). Such rapid enzymatic casein degradation in the small intestine is essential for fast insulin release and absorption.

### 3.2 Release Profile of CasNP/Insulin/C_10_ in GI Tract-Mimicking Conditions *In Vitro*

Since protein digestion mainly takes place between the stomach and jejunum,^54^ we investigated the enzymatic degradation of casNP/insulin/C_10_ in simulated gastric (1.0 mg/mL pepsin, pH 2.2), duodenal (0.02 mg/mL trypsin, pH 6.0), and jejunal fluids (0.1 and 0.7 mg/mL trypsin, pH 7.0, mimicking fasting and feeding conditions, respectively).^46,47^ After casNP/insulin/C_10_ was incubated in the simulated gastric, duodenal, and jejunal fluids for 15 to 60 min, SDS-PAGE analysis showed strong dark-colored casNP bands above the 250 kDa marker in gastric fluid (Figure 2A,B, in green dashed boxes), indicating that the casNP structure remained intact under the gastric environment. No free casein band below 37 kDa was detected in the gastric fluid, suggesting that most of the casein remained associated with the nanoparticle. To further distinguish the effects of acidity from enzymatic activity, casNP/insulin/C_10_ was also incubated at pH 2.2 in the absence of pepsin for 15 to 60 min. Under this condition, no casNP bands above 250 kDa were observed, while prominent protein bands appeared below 37 and around 10 kDa, indicating that acidic conditions destabilized the nanoparticles and led to casein and insulin release (Figure S2). In contrast, in the presence of pepsin at the same pH, strong casNP bands were maintained above 250 kDa with minimal degradation. The emergence of casein bands in the acidic-only condition can be attributed to the detachment of the casein layer under the electrophoresis condition. The observation of different SDS-PAGE patterns with and without pepsin can be attributed to the protective interaction between pepsin and the cross-linked casein coating, where pepsin molecules may bind to the casein surface, preventing acid-induced aggregation or electrophoresis-caused denaturation.

**Figure 2:**
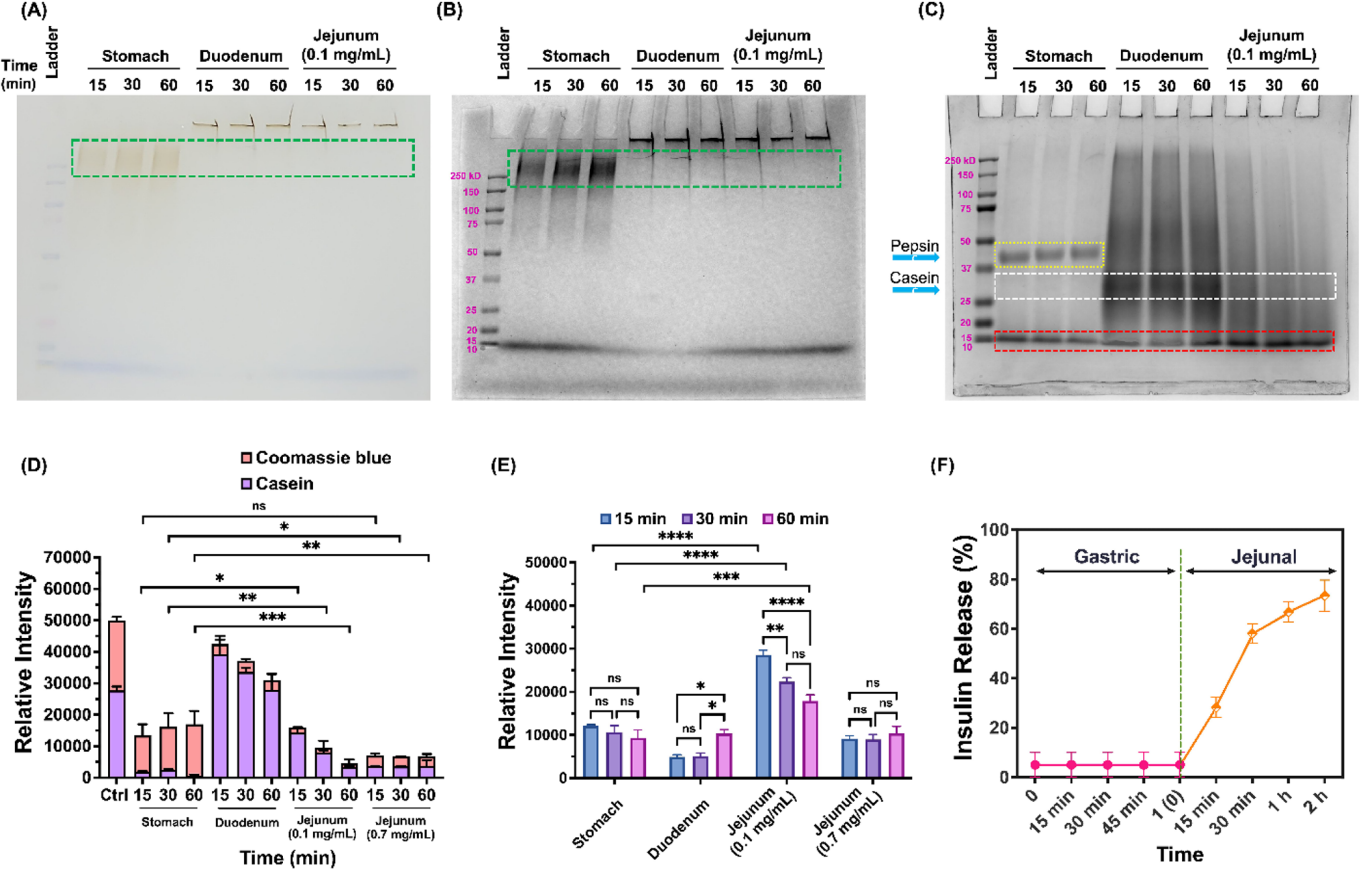
Stability of casNP/insulin/C_10_ in the gastric condition, and the enzymatic-triggered insulin release in the intestinal condition. Representative images of gel electrophoresis demonstrating the bands of (**A**) casNP/insulin/C_10_ without and (**B**) with Coomassie blue staining for proteins and (**C**) Coomassie blue-stained protein bands with molecular weights less than 50 kDa, after incubating casNP/insulin/C_10_ in the simulated gastric, duodenal, and jejunal fluids for 15, 30, and 60 min, respectively. (**D**) Quantified band intensities for undigested caseins (sum of those remaining on IONPs and those that fell off after electrophoresis) and (**E**) fragments of digested casein and insulin with molecular weights of 10 kDa in different conditions, based on the Coomassie blue staining of corresponding bands. (**F**) Insulin release profiles of casNP/insulin/C_10_ in simulated gastric and jejunal fluids were quantified based on the fluorescence signal intensities of FITC-insulin.

Bands below 37 kDa in the gels correspond to casein molecules detached from casNP/insulin/C_10_ under the electrophoresis conditions,^55,56^ as validated by the SDS-PAGE of PBS-treated casNP/insulin/C_10_ (Figure S2). This observation enables a comparative analysis of the casein layers after incubating casNP/insulin/C_10_ in different simulated fluids by assessing both the casein content retained on casNP/insulin/C_10_ and that which detached (Figure 2C, in white dashed boxes).

Quantitative analysis of the band intensities supported these observations. Gastric fluid showed lower detectable free casein (26.9 ± 4.5% to 34.0 ± 2.2% retention at 15 to 60 min) compared to duodenal fluid (85.0 ± 9.2% to 98.3 ± 8.1% retention) and untreated control. Jejunal fluid caused a marked reduction in intact casein in a trypsin concentration-dependent manner, with 0.1 mg/mL trypsin reducing retention to 32.1 ± 7%, 19.2 ± 6%, and 11.1 ± 4% at 15, 30, and 60 min, respectively, and 0.7 mg/mL trypsin further lowering retention to 13.5 ± 5%, 15.9 ± 2%, and 10.4 ± 1.4% (Figure 2D). The lower digestion of casein layers in the simulated duodenal fluids can be ascribed to the lower trypsin concentration and suboptimal pH for trypsin activity.^57,58^

This quantitative trend aligned with the SDS-PAGE analysis, where insulin and casein fragments near 10 kDa (Figure 2C, red dashed box) were observed under both jejunal conditions, with weaker band intensities at 0.7 mg/mL trypsin compared to 0.1 mg/mL. In contrast, these low-molecular-weight fragments were much more pronounced in the jejunal fluid than in the simulated gastric or duodenal fluids (Figure 2E). These results suggest a more efficient payload release from casNP/insulin/C_10_ in the jejunum, the preferred region of the GI tract for insulin absorption, than in the stomach and duodenum. It is worth noting that the intensities of bands for insulin and casein fragments at 10 kDa were significantly lower in the condition with 0.7 mg/mL trypsin compared to the other simulated jejunal conditions with less digestive capacity. This reduction is possibly due to the further proteolysis into ultralow-molecular-weight fragments that were not retained in the SDS-PAGE.

We further quantified insulin release under these conditions by measuring the fluorescence signal intensity from FITC using FITC-insulin encapsulated in the formulation. The results showed that the percentage of released insulin was 4.3 ± 2.8% in the simulated gastric fluid within 1 h, but sharply increased to 28.2 ± 4.1, 58.1 ± 4.0, 66.7 ± 4.1, and 73.3 ± 6.3% after incubating in the simulated jejunal fluid (0.7 mg/mL trypsin, pH 7.0) for 15, 30, 60, and 120 min, respectively (Figure 2F).

### 3.3 Biodistribution of CasNP/Insulin/C_10_

We labeled casNP/insulin/C_10_ with an NIR dye, NIR830, to visualize and quantify the casNP/insulin/C_10_ in the mouse GI tracts. Following oral gavage of NIR830-casNP/insulin/C_10_ at a dosage of 50 IU/kg of body weight, *ex vivo* NIR imaging was performed on collected organs and GI tissues at various time points. As shown in Figure 3A, NIR signals tracked the passage of NIR830-labeled casNP/insulin/C_10_ through the GI tracts from 15 min to 24 h postadministration. We estimated that the half gastric emptying time of casNP/insulin/C_10_ was <15 min, according to the radiance efficiency-based quantification of NIR830 signals showing <10.8 ± 1.7% of the administered casNP/insulin/C_10_ retained in the stomach at that time point (Figure 3C). It should be noted that NIR830 is conjugated to casein, and therefore, the observed signals mainly reflect the fate of casein rather than insulin or intact nanoparticles. Since nanoparticles are partially destabilized in the jejunal environment, NIR830 imaging may provide the early phase of the organ-specific biodistribution of intact casNP/insulin/C_10_. However, the systemic absorption of casNP/insulin/C_10_ and release of payload insulin need to be interpreted in conjunction with complementary measurements such as plasma insulin levels and iron content of organs. Quantifying NIR830 fluorescence signals further revealed that most casNP/insulin/C_10_ reached and then accumulated in the small intestine between 15 min and 8 h postoral gavage. Specifically, the percentage of total NIR830 signal localized in the small intestine was 76.1 ± 8.4% at 15 min, peaked at 86.9 ± 0.4% at 1 h, and then gradually declined to 65.7 ± 10.3% at 8 h, with no detectable signals at 24 h (Figure S3). We further investigated the distribution of casNP/insulin/C_10_ within the intestinal structures based on the NIR830 signals detected in excised small intestine tissues. At 1 h time point after oral administration of casNP/insulin/C_10_, NIR signals were primarily rising from the surfaces of the villi. The presence of casNP/insulin/C_10_ was confirmed by positive immunofluorescence staining of E-cadherin, as shown in the fluorescence image in Figure 3B. In addition, NIR signals were undetectable in the kidneys and spleen at all time points, while the level of NIR signal in the liver was ∼24.6 times lower than that observed in the small intestine (Figure 3C). To investigate whether oligosaccharide-coated IONPs were also absorbed in the small intestine after casNP/insulin/C_10_ was digested, we measured the Fe contents in the collected organs immediately after *ex vivo* imaging. The results indicated that Fe contents in these organs were not statistically significantly different from those of animals that did not take casNP/insulin/C_10_ (Figure 3D). However, it should be noted that there were significant variations in the iron measurement that affected the sensitivity of detecting the small changes in the Fe content of those organs. A larger sample size is needed in future studies to determine whether IONPs can be absorbed by the small intestine or other organs.

**Figure 3:**
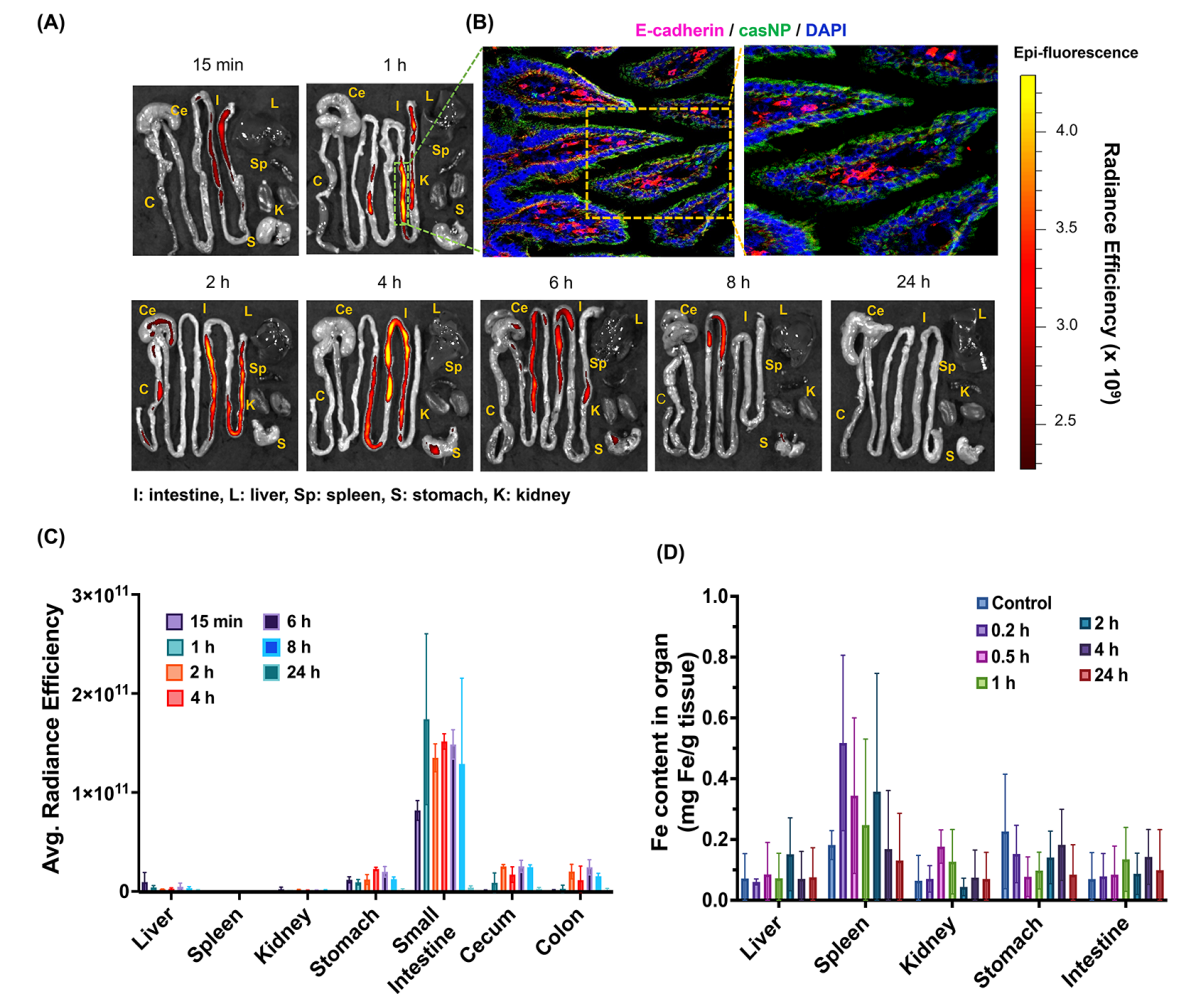
Biodistribution of casNP/insulin/C_10_ in diabetic mice. (**A**) Representative *ex vivo* fluorescence images of mouse organs collected at different time points after the oral gavage of NIR830-labeled casNP/insulin/C_10_. (**B**) A fluorescence image of NIR-positive small intestine tissues is used as an example to demonstrate the accumulation of NIR signals on the villi at all time points (scale bar: 100 μm). (**C**) ROI-based measurements of the radiance efficiencies of NIR signals and the percentages of signals distributed in the organs at different time points based on the *ex vivo* images. (**D**) Measurement of Fe contents in the collected organs for assessing the biodistribution of IONP cores after casNP/insulin/C_10_ was digested.

We also performed a comprehensive hematological and biochemical analysis of blood samples collected from the diabetic mice 2 h after oral gavage of casNP/insulin/C_10_ at 50 IU/kg body weight and PBS at an equal volume as a placebo control. The results showed no statistically significant difference between the casNP/insulin/C_10_ and placebo groups across a broad array of chemical and hematological markers in the blood (Table S2), including essential electrolytes such as sodium, potassium, and calcium; kidney function indicators such as BUN and CRE; liver function markers such as ALT, ALP and TBI; serum proteins such as total protein (TP), ALB, and GLOB, and PHOS; as well as digestive enzymes such as AMY. Levels of various blood cell components and hemoglobin parameters were also not affected, such as WBC, RBC, LYM, MON, NEU, HGB, HCT, MCV, MCH, MCHC, and PLT (Table S3). These findings indicate that casNP/insulin/C_10_ did not cause detectable acute toxicity at the tested dosage.

### 3.4 Bioavailability of Orally Delivered Insulin in Nondiabetic Mice

To evaluate the pharmacokinetics of oral versus subcutaneous insulin delivery in nondiabetic mice to provide the baseline for comparing the results obtained from diabetic models, two groups of healthy mice (N = 4/group, equal number of male and female) were administered casNP/insulin/C_10_ orally at 100 IU/kg or s.c. injection of IAsp at 5 IU/kg, respectively. In male mice receiving oral casNP/insulin/C_10_ (100 IU/kg), insulin concentrations rose rapidly from 1,538 μIU/mL at baseline to a peak of 2,286 μIU/mL at 15 min, followed by a moderate decline and sustained exposure through 8 h (1,467 μIU/mL). Female mice in the oral group exhibited slower absorption, with an initial decrease at 15 min (1,177 μIU/mL), followed by a gradual rise to a peak of 1,989 μIU/mL at 4 h and a slight decline to 1,732 μIU/mL at 8 h. In male mice receiving s.c. IAsp (5 IU/kg), insulin was high at baseline (2,053 μIU/mL), increased modestly at 15 min (2,429 μIU/mL), then declined to 1,500–1,650 μIU/mL over 1 to 4 h before rising slightly at 8 h (1,801 μIU/mL). Female s.c. IAsp-treated mice demonstrated a robust early response, peaking at 2,506 μIU/mL at 1 h, followed by a gradual decline, yet maintaining elevated levels at 8 h (1,942 μIU/mL) (Figure 4A). The relative insulin bioavailability for casNP/insulin/C_10_ was calculated to be 18.1% after combining the data of male and female mice. Collectively, these results indicate that oral casNP/insulin/C_10_ achieves systemic insulin exposure with sex-dependent kinetics, whereas s.c. IAsp provides consistently high and sustained insulin levels in both sexes.

**Figure 4:**
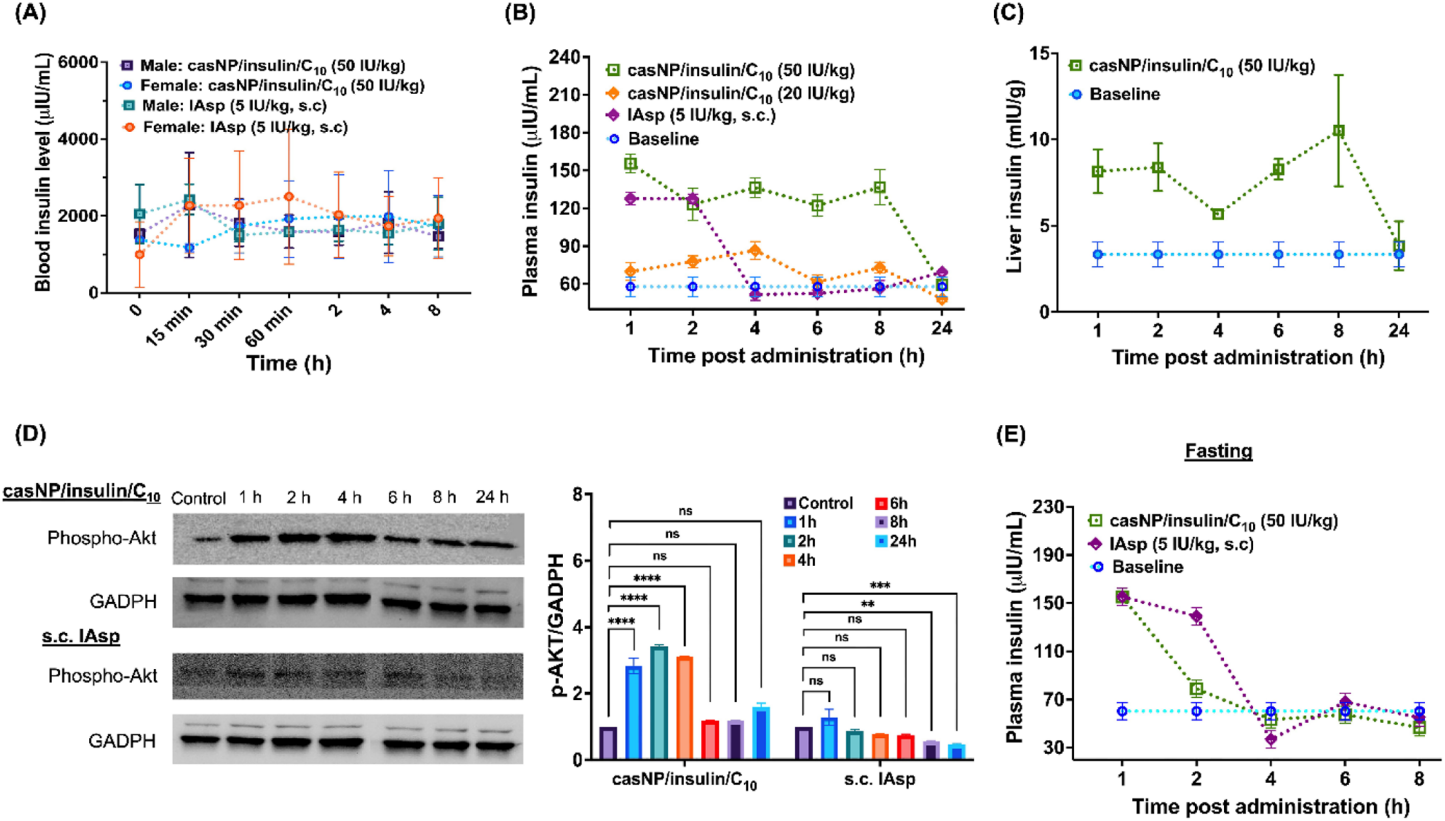
Insulin bioavailability in nondiabetic mice and in diabetic mice under fed and fasted conditions after oral casNP/insulin/C_10_ administration. (**A**) Blood insulin levels in nondiabetic mice before (0 h) and at 15, 30 min, 1, 2, 4, and 8 h after administration of casNP/insulin/C_10_ by oral gavage (50 IU/kg) or IAsp by s.c. injection (5 IU/kg). (**B**) Plasma insulin levels of diabetic mice with normal feeding conditions were measured from 1 to 24 h after oral gavage of casNP/insulin/C_10_ at 20 and 50 IU/kg body weight and s.c. injection of IAsp at 5 IU/kg body weight. The insulin levels in mice receiving no treatment were measured as the baseline. (**C**) Changes of insulin levels in the mouse livers 1 to 24 h after oral gavage of casNP/insulin/C_10_ at 50 IU/kg body weight. (**D**) Western blotting showing the levels of Akt phosphorylation in the livers of mice after receiving casNP/insulin/C_10_ and IAsp for 1, 2, 4, 6, 8, and 24 h. (**E**) Plasma insulin levels of fasted mice 1 to 8 h after the administration of casNP/insulin/C_10_ (50 IU/kg body weight, oral) and IAsp (5 IU/kg body weight, s.c.).

### 3.5 Pharmacokinetics of CasNP/Insulin/C_10_ in Diabetic Mice

To investigate the dosage-dependent effects and insulin bioavailability delivered via casNP/insulin/C_10_ in diabetic mice, we used two oral dosages, i.e., 20 and 50 IU/kg of body weight. We measured the plasma insulin levels at 1, 2, 4, 6, 8, and 24 h postadministration. CasNP/insulin/C_10_ was found to effectively increase the plasma insulin levels to 69.9 ± 4.9 and 155.47 ± 5.1 μIU/mL in 1 h at the dosages of 20 and 50 IU/kg body weight, respectively (Figure 4B), compared to the baseline of 57.8 ± 5.5 μIU/mL in the untreated mice. Importantly, the dosage-dependent elevation of insulin levels was maintained for 8 h with a single administration of casNP/insulin/C_10_ at both dosages. In comparison, IAsp, the standard-of-care treatment for diabetic conditions, was given to mice via s.c. injection at the dosage of 5 IU/kg body weight as a control, which boosted the plasma insulin levels to 127.6 ± 3.5 μIU/mL but persisted for only 2 h (Figure 4B). These results demonstrated that oral casNP/insulin/C_10_ at 50 IU/kg body weight offered comparable efficacy to IAsp (5 IU/kg body weight, s.c.) in elevating the plasma insulin levels of diabetic mice, but with a much longer-lasting effect (∼8 h).

Orally delivered insulin, after being absorbed by the GI tract, enters the portal vein and accumulates in the liver, which regulates the insulin levels in the plasma. Hence, we also measured the insulin levels in the livers from 1 to 24 h after the diabetic mice received oral gavage of casNP/insulin/C_10_ at 50 IU/kg body weight. The liver insulin levels were significantly elevated from 8.1 ± 0.8 to 10.5 ± 2.2 mIU/g tissue weight, as measured at 1 and 8 h after oral gavage of casNP/insulin/C_10_, while the baseline in untreated mice was 3.3 ± 0.5 mIU/g tissue weight (Figure 4C). The increase in hepatic insulin levels persisted for 8 h after oral gavage, which was consistent with the profile of plasma insulin levels. Elevated hepatic insulin levels at 8 h were also ∼76.9 times higher than those in the plasma, further confirming the regulatory role of the liver in the metabolism of insulin absorbed via the GI tract. To validate the hepatic accumulation of orally delivered insulin, we examined the levels of Akt phosphorylation in the mouse liver using Western blotting, as the phosphatidylinositol 3-kinase (PI3K) insulin signaling pathway can be triggered by the activation of insulin receptors after insulin binding.^59,60^ The results revealed a marked increase in Akt phosphorylation primed at 2 h postoral administration of casNP/insulin/C_10_ with a subsequent time-dependent decrease, as evidenced by the reduced band intensities (Figure 4D). In contrast, s.c. injected IAsp did not induce a noticeable activation of Akt phosphorylation, highlighting the hepatic targeting advantage of oral casNP/insulin/C_10_ delivery.

After confirming the liver accumulation of insulin delivered by casNP/insulin/C_10_, we next investigated whether casNP/insulin/C_10_ could overcome the risk of hypoglycemia under fasting conditions. Diabetic mice were fasted overnight before receiving casNP/insulin/C_10_ at 50 IU/kg body weight orally or IAsp at 5 IU/kg body weight s.c., respectively. The plasma insulin levels at different time points declined rapidly to the baseline within 2 h after oral gavage of casNP/insulin/C_10_. The distinct patterns of plasma insulin kinetics were noticed between fasted and fed diabetic mice receiving the same casNP/insulin/C_10_ dosage, indicating the capability of casNP/insulin/C_10_ to respond to food intake and prevent hyperinsulinemia under fasting conditions. On the other hand, plasma insulin levels in the IAsp-treated mice remained consistent regardless of feeding status, resembling the response seen in fed mice (Figure 4E). Taken together, these results highlighted that administration of oral casNP/insulin/C_10_ not only enhances insulin bioavailability and hepatic targeting but also offers a reduced risk of hypoglycemia by adapting insulin release in response to the metabolic demand in diabetic mice.

### 3.6 Efficacy of CasNP/Insulin/C_10_ in Controlling Glycemia

Diabetic mice were given casNP/insulin/C_10_ at dosages of 20 and 50 IU/kg body weight once per day at 8:30 am for 5 consecutive days to investigate the dosage-dependent effect. The efficacy of casNP/insulin/C_10_ was evaluated by measuring the mice’s BGLs immediately before and 1, 2, 4, 6, and 8 h after oral administration on Days 1, 2, and 5 (Figure 5A). The control groups included clinically used IAsp at 5 IU/kg body weight (s.c.) and PBS as the placebo. As shown in Figure 5B, casNP/insulin/C_10_ exhibited a significantly more effective BGL-lowering effect at the higher dosage (50 IU/kg body weight) on Days 1 and 2 compared to the lower dosage (20 IU/kg body weight). However, by Day 5, both dosages demonstrated comparable glycemic control, suggesting a potential cumulative effect over the treatment period. Notably, the BGL control efficiency of casNP/insulin/C_10_ at the higher dosage was comparable to that of s.c. IAsp but exhibited a longer-lasting effect, particularly on Days 2 and 5. At the lower dosage, casNP/insulin/C_10_ maintained the IAsp-comparable BGL-lowering effect for 2 h on Day 2, which was further prolonged on Day 5. In contrast, IAsp exhibited similar efficacy in controlling glycemia on Days 1, 2, and 5. These trends suggested that oral casNP/insulin/C_10_, particularly at 50 IU/kg, offered improved and sustained glycemic regulation compared to IAsp, likely due to enhanced hepatic insulin accumulation and regulated plasma insulinization by the liver. Interestingly, the convergence of efficacy between the two dosages on Day 5 indicated that lower dosages of casNP/insulin/C_10_ may exert efficacy with repeated dosing, possibly due to physiological adaptation, improved insulin sensitivity, or hepatic insulin storage. The observed dosage- and time-dependent response highlighted the need for dynamic dosage adjustment when using oral insulin formulations like casNP/insulin/C_10_, suggesting that casNP/insulin/C_10_ is a stable and effective oral insulin delivery method in liquid and drinkable form.

**Figure 5:**
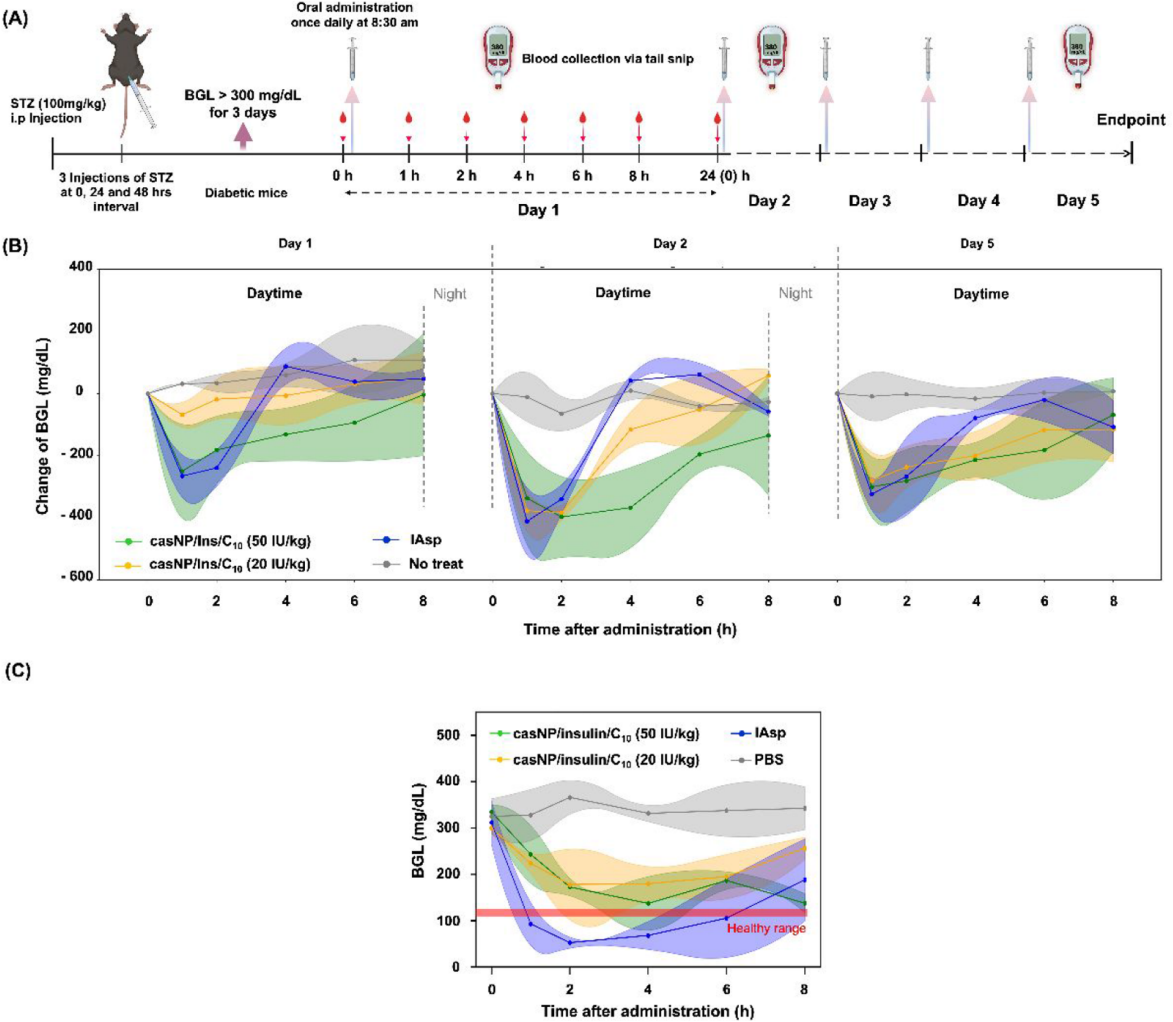
Efficacy of casNP/insulin/C_10_ in controlling the glycemia of diabetic mice. (**A**) Illustration of the study design. (**B**) Changes of BGLs in diabetic mice on Days 1, 2, and 5 after receiving casNP/insulin/C_10_ orally at 20 and 50 IU/kg body weight, IAsp s.c. at 5 IU/kg body weight, and PBS as a placebo, daily at 8:30 am. (**C**) BGLs of diabetic mice under fasting conditions were monitored for 8 h after administration of treatments and placebo.

To assess the risk of hypoglycemia induced by casNP/insulin/C_10_, diabetic mice were fasted overnight. The mice were then orally administered casNP/insulin/C_10_ at the two tested dosages (20 or 50 IU/kg of body weight). The control group received either an s.c. injection of IAsp (5 IU/kg) or oral PBS. BGL was monitored at 0 (predose), 1, 2, 4, 6, and 8 h postadministration to evaluate the hypoglycemic profile of the mice. As shown in Figure 5C, IAsp exhibited a prolonged BGL-lowering effect, with BGL decreasing slowly and gradually in mice receiving casNP/insulin/C_10_, as BGL stayed above the healthy range of ∼50 mg/dL. However, IAsp-treated mice showed a sharp decrease in BGL at 1 h postinjection, reaching 109.5 ± 3.2 mg/dL, which is below the normoglycemic threshold of 120 mg/dL,^61^ indicating a risk of hypoglycemia.

## 4 Discussion

In this study, a drinkable nanoformulation of oral insulin was developed by encapsulating insulin and permeation enhancer C_10_ onto milk protein casein cross-linked on the surface of an oligosaccharide-coated IONP. After validating the gastric stability and intestinal enzyme-triggered payload release of the developed casNP/insulin/C_10_ *in vitro* using simulated gastric and intestinal fluids, *in vivo* studies on an STZ-induced diabetic mouse model were performed, which revealed a fast gastric emptying of casNP/insulin/C_10_ with a half-emptying time of <15 min, a quick and long-lasting insulinization in the liver and plasma from 1 to 8 h after oral gavage, and an insulin bioavailability of 8.3%. The efficacy studies showed that casNP/insulin/C_10_ provided longer glycemic control and less risk of hypoglycemia than s.c.-injected IAsp for diabetic mice. Compared to the enteric coatings used by current oral insulin formulations, caseins are not only abundant milk proteins in our daily diets, but also natural carriers for a variety of biomolecules, including C_10_.^62^ Using cross-linked caseins as the outer layer, casNP/insulin/C_10_ may avoid the side effects encountered by other conventional oral insulin formulations using synthetic enteric coatings,^63^ thus offering a more favorable biosafety profile. CasNP/insulin/C_10_ with a hydrodynamic size of ∼25 nm, is fully dissolved in water and is resistant to gastric degradation, therefore exhibiting a rapid gastric emptying with a half-emptying time similar to liquid (∼13 ± 1 min).^64^ This feature allows casNP/insulin/C_10_ to reach the small intestine much faster and more efficiently than capsule or tablet formulations that may stay in the stomach for several hours,^65^ thus enabling a shorter time of action.

The reported casNP/insulin/C_10_ is prepared as a drinkable liquid formulation, which offers the advantages of being easy to use and improving patient compliance, especially for pediatric patients. For future translation, its long-term stability should be established. Preliminary studies indicate that the prepared casNP/insulin/C_10_ remains physically stable for at least 1 year at 4 °C, with minimal aggregation. Future studies will focus on evaluating functional stability, developing scalable and pharmaceutical manufacturing protocols,^66^ and optimizing shelf life to facilitate clinical translation.

In this context, oral insulin formulations investigated in clinical trials, e.g., ORMD-0801, I338, Tregopil, and Capsulin, are mainly enteric-coated capsules or tablets.^14,67–69^ The enteric coatings are sensitive to given pH ranges, which may vary greatly among individuals at different times of the day,^70^ causing suboptimal efficiencies of insulin delivery and absorption in the desired regions of the GI tract.^71^ This issue is compounded by the pH-dependent solubility of insulin, which increases from 0.14 mg/mL at pH 6.0 to 0.70 mg/mL at pH 6.7.^72^ Payload release from casNP/insulin/C_10_ is primarily facilitated by the intestinal enzyme trypsin through the breakdown of the casein layer, which occurs preferentially in the jejunum with the optimal pH but not the duodenum.^57,58^ Insulin release in the jejunum instead of the duodenum is particularly advantageous, not only because of the higher insulin solubility in the basic jejunal condition but also because of the substantially larger surface area of the jejunum, with the villi and circular folds specialized in nutrient absorption. By achieving small intestine-targeted release of insulin, casNP/insulin/C_10_ provides a much-improved insulin bioavailability of 8.3%, compared to other oral insulin formulations with <1%.^11,12^

Notably, the profiles of mouse plasma insulin levels and BGLs after receiving IAsp are likely attributed to the insulin clearance from the subcutaneous depot, in contrast to the physiologically relevant hepatic first-pass metabolism achieved with casNP/insulin/C_10_. Fasted mice treated with IAsp showed a sharp decrease in BGLs at 1 h postinjection that falls below the normoglycemic threshold, indicating a risk of hypoglycemia.^73^ This rapid reduction is due to the immediate systemic exposure of insulin-sensitive tissues, such as skeletal muscle and adipose tissue, to high insulin levels, bypassing the liver, which plays a key regulatory role in glucose metabolism.^74^ In contrast, mice receiving casNP/insulin/C_10_ showed a mild decrease in BGLs that was maintained above the healthy range, thus reducing the hypoglycemic risk. This feature is likely attributed to the hepatic first-pass pathway, whereby orally delivered casNP/insulin/C_10_ is absorbed via the intestinal epithelium into the portal circulation, facilitating preferential insulin delivery to the liver before absorbing into the peripheral tissues. As a result, insulin is first used to suppress hepatic glucose output and promote glycogen synthesis, providing a physiologically prioritized “buffering step.” This prevents the abrupt, high systemic insulin peaks characteristic of subcutaneous delivery and instead produces a slower, liver-first insulinization profile. Such a profile more closely mimics endogenous postprandial insulin kinetics and therefore offers a safer pharmacodynamic response. By preventing sudden spikes in peripheral insulin exposure, the casNP/insulin/C_10_ formulation avoids the exaggerated glucose uptake by muscle and adipose tissue that can lead to hypoglycemia after s.c. IAsp administration. This distinction, fast hepatic engagement followed by more gradual peripheral exposure, represents a major therapeutic advantage of the oral route, providing a more physiological and safer insulin profile.^75,76^

Notably, our current formulation uses IONP as a scaffold to assemble caseins for a core–shell structure. In this case, the fate of iron from orally administered IONPs may follow three different mechanisms. With approximately 2.5 mg of iron element in the high dose of 50 IU/kg casNP/insulin/C_10_, a fraction of the iron released from IONPs may be excreted via feces, whereas the intestine has the capability to absorb the nonheme form of iron. Another part of iron from IONP, following partial dissolution in the mildly acidic duodenum, may cross enterocytes via the luminal membrane-expressed iron importer DMT1 (divalent metal transporter 1).^77^ Inside enterocytes, iron can enter the labile iron pool and is either stored as ferritin or trafficked to the basolateral membrane for export via ferroportin, the essential gatekeeper that also mediates the systemic entry of nanoparticulate-derived iron.^78^ During export, ferrous (Fe^2+^) is oxidized to ferric (Fe^3+^) by ferroxidases and rapidly chelated by apotransferrin, forming circulating transferrin–iron complexes that are delivered to tissues and hepatocytes. Importantly, like dietary iron, the absorption of iron released from IONPs is tightly controlled by the hepcidin–ferroportin axis, a mechanism that limits excessive systemic accumulation of iron.^79,80^ Additionally, some of the IONPs can cross the intestinal epithelium through transepithelial or paracellular routes. Gu et al. systematically compared IONPs with different core sizes (i.e., 5, 15, and 25 nm) but the same polymer coatings. IONPs with a 5 nm core size were rapidly cleared through the kidney, while larger IONPs with 15 and 25 nm core sizes were primarily sequestered by hepatic Kupffer cells and splenic macrophages. Within these cells, lysosomal degradation liberated iron, which was efficiently incorporated into ferritin and trafficked via the transferrin-mediated systemic iron cycle. Notably, larger IONPs exhibited slow yet complete metabolic turnover without persistent tissue accumulation.^81^ Taken together, the iron-containing casNP with the doses used in this work will unlikely lead to significant systemic iron overload. Nevertheless, the IONP core can be replaced by other nonmetal nanoparticles, such as carbon nanodots, to mitigate the concern over potential iron-induced toxicity.

## 5 Conclusions

We reported a milk protein-based nanoformulation casNP/insulin/C_10_ as a long-acting oral insulin delivery system. The developed formulation not only protected the encapsulated insulin and permeation enhancer C_10_ from gastric degradation but also enabled efficient intestinal enzyme-triggered release of the payload *in vitro*. In diabetic mice, casNP/insulin/C_10_ demonstrated rapid gastric emptying and efficient intestinal delivery, and C_10_-facilitated effective absorption of insulin in the small intestine through the villi resulting in increased insulin levels in both liver and plasma and decreased BGLs. CasNP/insulin/C_10_ demonstrated a similar but much-extended efficacy in glycemic control compared to the clinically used subcutaneous IAsp, while avoiding hypoglycemia in fasted mice treated with IAsp. The nanoformulation was well tolerated, with no observable toxicity. Together, these results indicate that the developed casNP/insulin/C_10_ holds promise in rendering an effective and drinkable oral insulin formulation for managing diabetes.

## Supplementary Material



## Data Availability

Data will be made available upon reasonable request.

## Notes

Characterization of casNP using TEM, DLS, zeta potential, and UV–visible spectrum of casNP and casNP/insulin/C_10_ (Figure S1); SDS-PAGE showing the stability of casNP/insulin/C_10_ in gastric conditions and enzyme-triggered insulin release in intestinal conditions (Figure S2); e*x vivo* study of the percentage radiance efficiency of NIR signals in organs over time (Figure S3); physicochemical characteristics of casNPs (Table S1); levels of liver function markers in the blood of diabetic mice (Table S2); levels of blood cell components and hemoglobin in diabetic mice (Table S3) (PDF)
