# Supplementary material for: Small Intestine-Targeted Long-Acting Oral Insulin Formulation Based on Engineered Milk Protein Nanoparticles
Source: ACS Appl Bio Mater. 2025 Dec 24;9(2):1083–97. doi: 10.1021/acsabm.5c02114 (PMC12820955; doi:10.1021/acsabm.5c02114)
Supplement: Supplementary file 1 [file mt5c02114_si_001.pdf]

Supporting Information

Small Intestine Targeted Long-Acting Oral Insulin Formulation Based on Engineered Milk Protein Nanoparticles

Anbu Mozhi Thamizhchelvan[a], Yuancheng Li[a,b,], Jonathan Padelford[b], Ce Yang[b], Chunhua Yang[c], Peijian He[d], Ashan Galhena[a], Tianhe Wu[a], Malgorzata Lipowska[a], and Hui Mao[a,]**

[a]*Department of Radiology and Imaging Sciences, Emory University School of Medicine, Atlanta, Georgia 30322, United States of America*

[b]*5M Biomed, LLC, Atlanta, Georgia 30303, United States of America*

[c]*Institute for Biomedical Sciences, Georgia State University, Atlanta, Georgia 30303, United States of America*

[d]*Department of Medicine, Emory University School of Medicine, Atlanta, Georgia 30322, United States of America*

*Corresponding Authors: Hui Mao, PhD, Department of Radiology and Imaging Sciences, Emory University School of Medicine, Atlanta, GA 30322, USA, *Email address:* hmao@emory.edu; Yuancheng Li, 5M Biomed, LLC, Atlanta, GA 30303, USA, *Email address:* yli_5mbiomed@hotmail.com

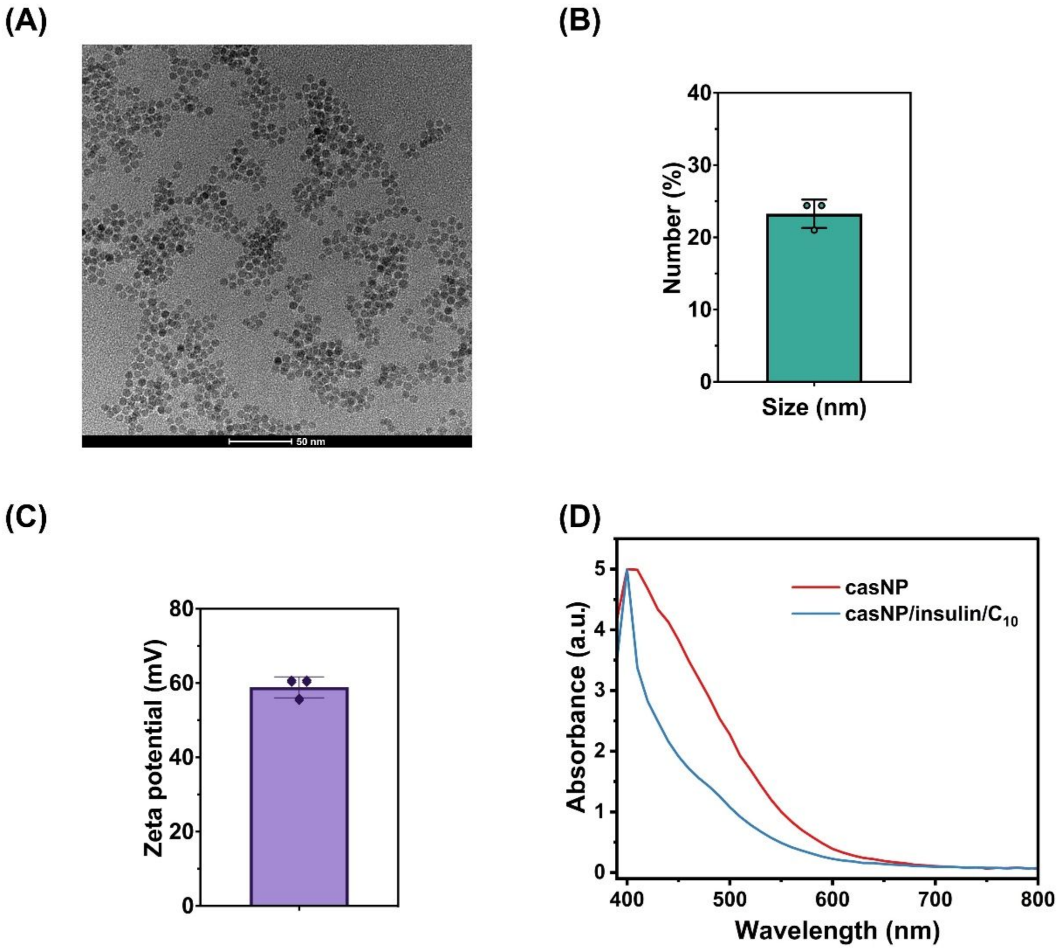

Figure S1. Characterization of casNP (A) TEM image, (B) dynamic light scattering (DLS) showing the hydrodynamic size, (C) zeta potential, and (D) UV-visible spectrum of casNP and casNP/insulin/C_{10}

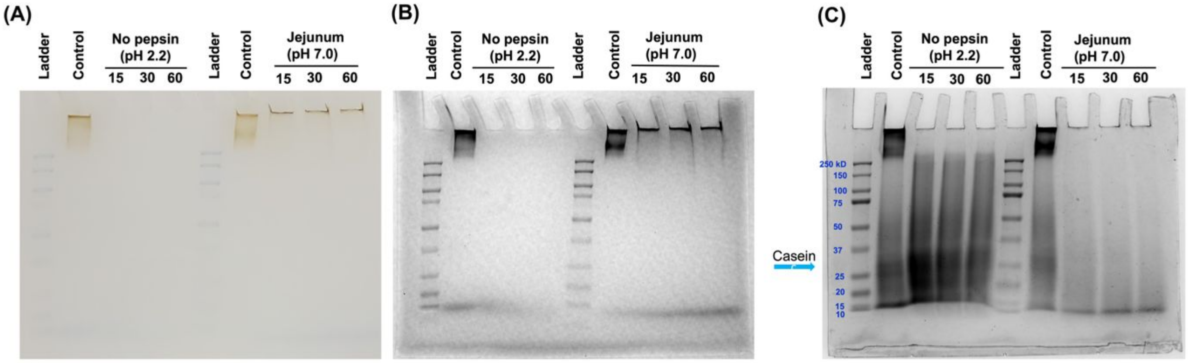

Figure S2. Stability of casNP/insulin/C_{10} in the gastric condition, and the enzymatic triggered insulin release in the intestinal condition. Representative images of gel electrophoresis demonstrating the bands of (**A**) casNP/insulin/C_{10} without and (B) with Coomassie blue staining for proteins and (**C**) Coomassie blue stained protein bands with molecular weight less than 50 kDa, after incubating casNP/insulin/C_{10} in PBS (pH 7.4; control), McIlvaine buffer at pH 2.2 (no pepsin), and jejunal fluids for 15, 30 and 60 min, respectively.

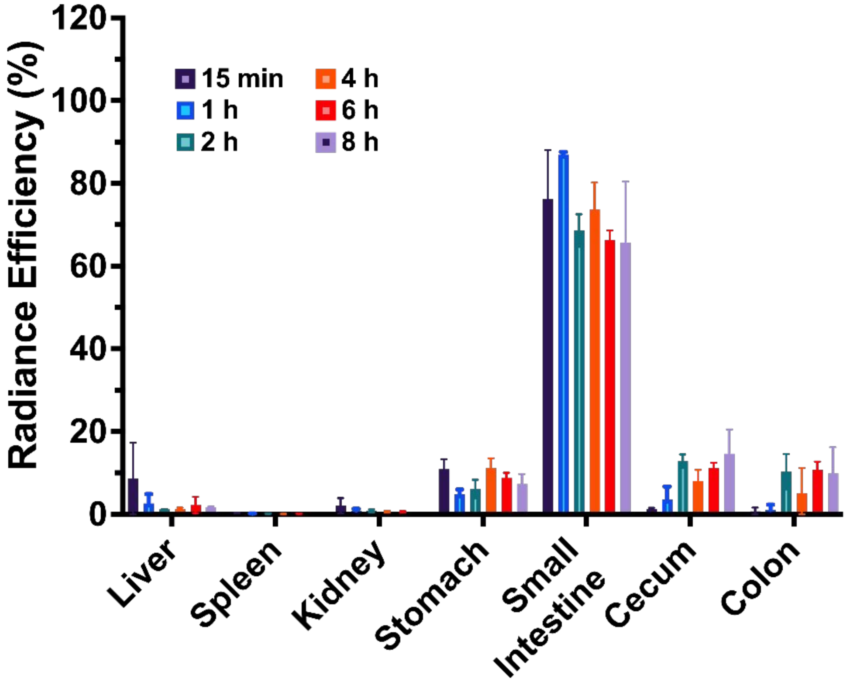

Figure S3. ROI-based measurements of the percentage radiance efficiencies of NIR signals distributed in the organs at different time points based on the *ex vivo* images.

Table S1: Physicochemical characteristics of casNPs

Core size	Casein/IONP (wt)	Casein/IONP (wt%)	Hydrodynamic diameter (nm)	ζ-potential (mV)
5 nm	1:1	34 ± 2	10.5 ± 1.1	-55.6 ± 4.9
	3:1	62 ± 7	18.1 ± 1.9	-45.6 ± 4.1
	5:1	81 ± 14	23.3 ± 1.9	-42.3 ± 2.9
	10:1	82 ± 14	25.7 ± 2.2	-61.6 ± 5.3
20 nm	1:1	33 ± 4	26.6 ± 1.8	-58.8 ± 3.1
	3:1	42 ± 7	36.5 ± 3.3	-66.2 ± 3.3
	5:1	76 ± 16	47.3 ± 4.2	-62.3 ± 2.9
	10:1	81 ± 26	63.4 ± 6.6	-73.6 ± 2.8

Table S2. Levels of liver functional markers measured in blood samples collected from diabetic mice in the control and casNP/insulin/C_{10} treated group

Analytes*	casNP/insulin/C_{10} Treated	Control
**GLOB (g/dL)**	1.4 ± 0.0	1.1 ± 0.1
**TP (g/dL)**	5.6 ± 0.0	5.1 ± 0.6
**K^+ (mM)**	5.8 ± 0.5	4.8 ± 1.0
**Na^+ (mM)**	145 ± 5	135 ± 18
**CRE (mg/dL)**	0.3 ± 0.0	0.3 ± 0.0
**PHOS (mg/dL)**	7.0±0.2	6.5 ± 1.4
**CA (mg/dL)**	10.2 ± 0.1	8.8 ± 1.3
**BUN (mg/DL)**	20.5 ± 2.1	22.5 ± 0.7
**TBIL (mg/DL)**	0.3 ± 0.0	0.3 ± 0.0
**AMY (U/L)**	753 ± 91	682 ± 75
**ALT (U/L)**	53.3 ± 4.8	47.6 ± 0.7
**ALP (U/L)**	56.0 ± 0.5	53.5 ± 9.2
**ALB (g/dL)**	4.2 ± 0.1	3.9 ± 0.4

*Abbreviations for the measured analytes are as following: ALB: albumin; ALP: alkaline phosphatase; ALT: alanine aminotransferase; AMY: amylase; TBIL: total bilirubin; BUN: blood urea nitrogen; CA: calcium; PHOS: phosphorus; CRE: creatinine; Na^+: sodium; potassium (K^+): TP total protein; GLOB: globulin;

Table S3. Levels of blood cell components and hemoglobin measured in blood samples collected from diabetic mice in the control and casNP/insulin/C_{10} treated group

Cells*	casNP/insulin/C_{10} Treated	Control
**PLT (10^9/I)**	369 ± 12	352 ± 46
**MCHC (g/dL)**	38.6 ± 0.3	38.3 ± 0.6
**MCH (pg)**	15.3 ± 0.4	15.3 ± 0.4
**MCV (FL)**	40.6 ± 0.7	40.0 ± 1.4
**HCT (%)**	37.3 ± 0.0	35.3 ± 0.7
**HGB (g/dL)**	14.3 ± 0.1	13.5 ± 0.2
**RBC (10^{12}/I)**	9.4 ± 0.2	8.8 ± 0.3
**NEU (10^9/I)**	0.6 ± 0.2	0.5 ± 0.1
**MON (10^9/I)**	0.3 ± 0.1	0.3 ± 0.2
**LYM (10^9/I)**	4.3 ± 0.9	5.1 ± 1.7
**WBC (10^9/I)**	6.5 ± 0.2	7.2 ± 0.2

*Abbreviations for the measured cells are as following: WBC: white blood cells; LYM: lymphocytes; MON: monocytes; NEU: neutrophils: RBC: red blood cells; PLT: platelets; HGB: hemoglobin; HCT: MCV: hematocrit; mean corpuscular volume; MCH: mean corpuscular hemoglobin; MCHC: mean corpuscular hemoglobin concentration;
